# Caffeine increases performance and leads to a cardioprotective effect during intense exercise in cyclists

**DOI:** 10.1038/s41598-021-03158-2

**Published:** 2021-12-21

**Authors:** Felipe Sampaio-Jorge, Anderson Pontes Morales, Rafael Pereira, Thiago Barth, Beatriz Gonçalves Ribeiro

**Affiliations:** 1 Higher Institutes of Education of CENSA (ISECENSA), Campos dos Goytacazes, Rio de Janeiro, 28030-260 Brazil; 2 Macaé Sports Secretary, City Government of Macaé (PMM), Macaé, Rio de Janeiro, 27913-080 Brazil; 3 Present Address: Laboratory Research and Innovation in Sports Sciences, Federal University of Rio de Janeiro (UFRJ), Macaé Campus, 50, Aluízio da Silva Gomes, Granja Dos Cavaleiros, Rio de Janeiro, 27930-560 Brazil; 4 Postgraduate Program in Nutrition, Josué de Castro Nutrition Institute, Federal University of Rio de Janeiro (UFRJ), Macaé, Rio de Janeiro, 21941-590 Brazil; 5 Integrative Physiology Research Center, State University of Southwest Bahia (UESB), Jequié, BA 45210-506 Brazil; 6 Laboratory of Bioactive Products, Federal University of Rio de Janeiro (UFRJ), Macaé, Rio de Janeiro, 27933-378 Brazil

**Keywords:** Circulation, Metabolism, Biochemistry, Cardiology

## Abstract

The present study was designed to investigate the effects of different caffeine dietary strategies to compare the impact on athletic performance and cardiac autonomic response. The order of the supplementation was randomly assigned: placebo(4-day)-placebo(acute)/PP, placebo(4-day)-caffeine(acute)/PC and caffeine(4-day)-caffeine(acute)/CC. Fourteen male recreationally-trained cyclists ingested capsules containing either placebo or caffeine (6 mg kg^−1^) for 4 days. On day 5 (acute), capsules containing placebo or caffeine (6 mg kg^−1^) were ingested 60 min before completing a 16 km time-trial (simulated cycling). CC and PC showed improvements in time (CC vs PP, Δ − 39.3 s and PC vs PP, Δ − 43.4 s; *P* = 0.00; ƞ^2^ = 0.33) and in output power (CC vs PP, Δ 5.55 w and PC vs PP, Δ 6.17 w; *P* = 0.00; ƞ^2^ = 0.30). At the final of the time-trial, CC and PC exhibited greater parasympathetic modulation (vagal tone) when compared to the PP condition (*P* < 0.00; ƞ^2^ = 0.92). Our study provided evidence that acute caffeine intake (6 mg∙kg^−1^) increased performance (time-trial) and demonstrated a relevant cardioprotective effect, through increased vagal tone.

## Introduction

Caffeine (1,3,7-Trimethylxanthine) is one of the most consumed supplements by both, general and athletes population^1^ owing to an expected ergogenic effect for endurance exercise^2^, anaerobic-based exercise^3^ and strength/power activities. However, regarding the caffeine supplementation for ergogenic aims, two concerns have been raised: (1) as it is described many mechanisms involving the autonomic nervous system (at central and peripherical sites), the cardiovascular safety of supplementation previously to exercises with high cardiovascular demands need to be investigated; (2) as for any drug, tolerance could be developed with a regular use, which have been discussed in recent studies^4,5^, but with contradictory results.

Despite caffeine (CAF) consumption is safe even in patients with cardiovascular disease^6^, its security is linked to the dose, as well as to the age, sex, pathophysiology of the consumer, or type of sport (if used for ergogenic aims). Indeed, CAF supplementation could promotes tachycardia^7^ and decreased perceived exertion, increasing the time until muscle fatigue during exercises^8^. Among proposed action mechanisms that explain how CAF influences cardiovascular function, the first one is the sympathetic autonomic nervous system activation by catecholamines released in blood plasma and the second is the blocking of the adenosine receptors (A1, A2a, A2b) on the central nervous system^9^. A primary role of adenosine in the nervous system appears to be to inhibit the release of various neurotransmitters, and possibly glutamate in particular, through presynaptic receptors. Therefore adenosine antagonists, such as CAF, can be expected to increase the release of neurotransmitters^10^. Although innocuous, these changes may be related to high risks of cardiovascular events^11^ especially during/after exercise^12^.

Findings from CAF use are focusing directly on heart cells and vessels, as a stimulant to induce arrhythmias through ryanodine receptors and their mutated forms. Caffeine, by binding to the ryanodine receptor, stimulates calcium fluxes that can result in arrhythmias, which are linked to the positive inotropic effect of caffeine. Another possibility is inhibition of phosphodiesterases, which results in an increase in the intracellular concentration of cAMP and cGMP (which under normal circumstances are degraded by phosphodiesterase)^13^.

The analysis of successive RR intervals (the time elapsed between two successive R-waves of the QRS signal on the electrocardiogram), also reported as heart rate variability (HRV), allows estimating the autonomic nervous system (ANS) activity through a non-invasive method^14^. Previous studies^15,16^ investigated the impact of CAF on ANS through HRV, reporting HRV decrease with some doses, while different strategies could increase the HRV. Methodological aspects could explain divergent results, especially owing to complex HRV behavior during exercise, which could be minimized with appropriate mathematical methods to extract RR interval variability, as with non-linear methods^17^. Notwithstanding, lack of pattern of supplementation approach, including dose and selected strategy, also contribute to divergence in the issue of this study.

Different CAF dietary strategies are used to enhance performance^18^. Some authors defend its use a high dose only on the competition’s day, after a withdrawal from any CAF source during the training period, since it is hypothesized that this strategy could enhance its ergogenic effect^19–21^. On the other hand, despite the findings of studies^22–24^ that indicate tolerance to ergogenic effects with the regular use of CAF, other authors^4,5^ recommend the use of doses that represent an average amount of caffeine above the usual (i.e., reducing the tolerance effect), having its ergogenic effect potentiated. Furthermore, findings^25^ indicate that doses of 300–400 mg of caffeine delay post-exercise parasympathetic reactivation, whereas others induced no caffeine effect at doses associated with < 3 mg kg^−1^ body mass. Although it is difficult comparing and contrasting the results, these inconsistent findings may be related to the dosage of caffeine used.

However, the use of these strategies can cause a high risk of adverse effects^4,5^, such as changes in autonomic modulation, which can promote an enabling environment for the development of rhythm disturbances and abnormal responses of heart rate^26^.

The present study was designed to investigate, in cyclists, the effects of different CAF dietary strategies to compare the impact on athletic performance and cardiac autonomic response.

## Results

### Food consumption analysis

Food intake did not exhibit significant main effects in treatment 24 h before the trials: energy (Kcal) PP = 2132 ± 743, PC = 2056 ± 389, CC = 2056 ± 389 (*P* = 0.45); carbohydrates (g day^−1^) PP = 250 ± 67, PC = 242 ± 67, CC = 276 ± 80 (*P* = 0.34); protein (g day^−1^) PP = 121 ± 45, PC = 114 ± 42, CC = 126 ± 47 (*P* = 0.1450); and lipids (g day^−1^) PP = 78 ± 45, PC = 69 ± 25, CC = 62 ± 27 (*P* = 0.17).

### Blinding efficacy

At the post-exercise assessment, in the PP condition, 7.1% (1) got their caffeine intake right, 14.3% (2) got it wrong, and 78.6% (11) were unable to answer. In the PC condition, 14.3% (2) were right, 14.3% (2) were wrong and 71.4% (10) were unable to answer. In the CC condition, 14.3% (2) were right, 7.1% (1) were wrong and 78.6% (11) were unable to answer.

### Blood caffeine concentration

The Blood sample analysis exhibited significant time (*P* = 0.0001) and treatment (*P* = 0.0001) main effects for caffeine concentration (Table 1).

**Table 1:** Analysis of blood caffeine concentration (n = 14).

	Baseline (µg/mL^−1^)	60’ post caffeine ingestion (µg/mL^−1^)
PP	0.30 (0.44)	0.30 (0.44)
PC	0.00 (0.32)	7.60 (1.32)*^#^
CC	0.45 (0.41)	8.19 (0.76)*^#^

Median (interquartile deviation) of caffeine concentration with 3 experimental conditions: Placebo–Placebo (PP), Placebo–Caffeine (PC) and Caffeine–Caffeine (CC).

*Significantly different from baseline (*P* < 0.05). ^#^Significantly different from PP (*P* < 0.05).

### HRV evaluation in the supine position

**Figure 1:**
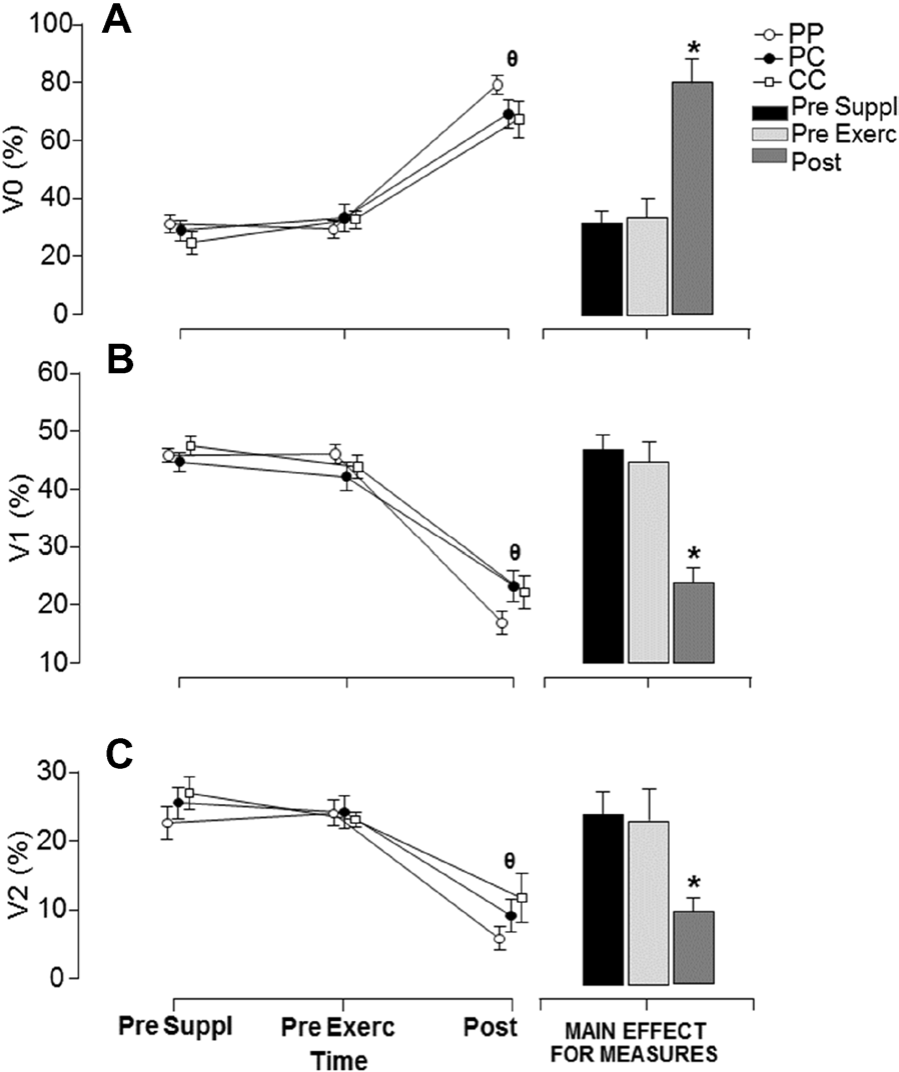
(**A**) Mean ± SE of V0 (%) at the pre supplementation, pre exercise, and post exercise (i.e., TT-test) with 3 experimental conditions: Placebo-Placebo (PP), Placebo-Caffeine (PC) and Caffeine-Caffeine (CC). (θ) Post was significantly different from other moments. (*) Significantly different from other moments (*P* < 0.05). (**B**) Mean ± SE of V1 (%) at the PRE suppl, PRE exerc and POST exercise (i.e., TT-test) with 3 experimental conditions: Placebo-Placebo (PP), Placebo-Caffeine (PC) and Caffeine-Caffeine (CC). (θ) Post was significantly different from other moments. (*) Significantly different from other moments (*P* < 0.05). (**C**) Mean ± SE of V2 (%) at the PRE suppl, PRE exerc and POST exercise (i.e., TT-test) with 3 experimental conditions: Placebo-Placebo (PP), Placebo-Caffeine (PC) and Caffeine-Caffeine (CC). (θ) Post was significantly different from other moments (*P* < 0.05). (*) Significantly different from other moments (*P* < 0.05).

No significant main effect was observed for treatment in all studied HRV parameters (V0—sympathetic modulation: F = 1.3, *P* = 0.293; ƞ^2^ = 0.106; V1—sympathetic and parasympathetic modulation: F = 0.471, *P* = 0.630; ƞ^2^ = 0.041; V2—parasympathetic modulation: F = 1.849, *P* = 0.181; ƞ^2^ = 0.144), indicating the supplementation strategy had no effect for comparisons from recording at supine position. On the other hand, a significant main effect for measures (V0: F = 491.36, *P* < 0.001; ƞ^2^ = 0.978; V1: F = 1015.27, *P* < 0.001; ƞ^2^ = 0.989; V2: F = 127.3, *P* < 0.001; ƞ^2^ = 0.920). A post hoc analysis indicated that all studied HRV parameters changed significantly after the TT-test, without differences between pre supplementation and pre exercise measures (Fig. 1).

Considering the caffeine supplementation comparisons, Bayesian analysis indicated moderate to anecdotal posterior probability favoring the null hypothesis, which was found for V0, V1 and V2. The results from inferential and Bayesian statistics are presented in Table 2.

**Table 2:** Bayesian analysis for the comparison of symbolic analysis parameters obtained at supine position and at different caffeine supplementation conditions.

Experimental condition		Prior odds	Posterior odds	BF_10, U_	Probability %
**V0**					
PP	PC	0.587	5.36	0.267^m^	21.1
PP	CC	0.587	12.20	0.537^a^	34.9
PC	CC	0.587	0.11	0.210^m^	17.4
**V1**					
PP	PC	0.587	0.25	0.185^m^	15.6
PP	CC	0.587	0.12	0.280^m^	21.9
PC	CC	0.587	0.26	0.218^m^	17.9
**V2**					
PP	PC	0.587	2.23	0.437^a^	30.4
PP	CC	0.587	23.00	0.647^a^	39.4
PC	CC	0.587	0.19	0.200^m^	16.7

The posterior odds have been corrected for multiple testing by fixing to 0.5 the prior probability that the null hypothesis holds across all comparisons (Westfall, Johnson, & Utts, 1997). Individual comparisons are based on the default *t* test with a Cauchy (0, r = 1/sqrt(2)) prior. The “U” in the Bayes factor denotes that it is uncorrected (PP: Placebo–Placebo; PC: Placebo–Caffeine; CC: Caffeine–Caffeine). Letters indicate the outcome classified as: A = anecdotal; M = moderate; V = very strong; E = extreme favoring the alternative hypothesis; a = anecdotal; m = moderate favoring the null hypothesis.

### TT-test time and average power output

**Figure 2:**
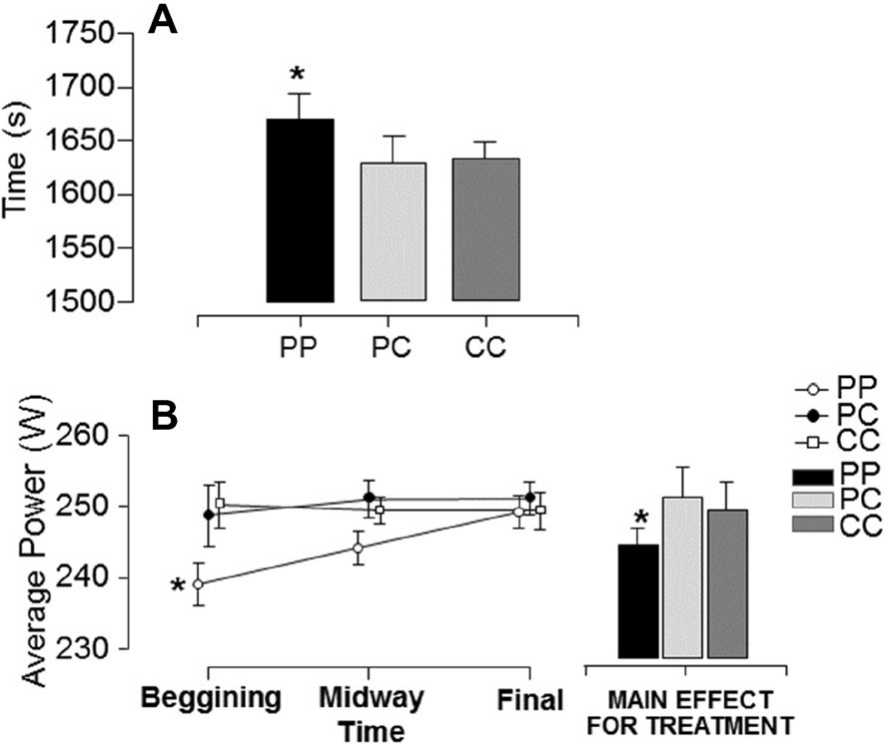
(**A**) Mean ± SE of time to execute the TT-test with 3 experimental conditions: Placebo-Placebo (PP), Placebo-Caffeine (PC) and Caffeine-Caffeine (CC). (*) Significantly different from other experimental conditions (*P* < 0.05). (**B**) Mean ± SE of average power output (W) with 3 experimental conditions: Placebo-Placebo (PP), Placebo-Caffeine (PC) and Caffeine-Caffeine (CC) in three different moments of the TT-test. (*) Significantly different from other experimental conditions (*P* < 0.05).

The TT-test time in seconds (s) exhibited a significant main effect for treatment (F = 6.49, *P* = 0.005; ƞ^2^ = 0.333). The time was significantly greater (*P* < 0.05) in the condition PP when compared to other conditions (Δ for PP vs PC was 43.4 s and for PP vs CC was 39.3 s). There was no difference (*P* > 0.05) between PC and CC (Δ for PC vs CC was − 4.07 s) (Fig. 2A). The average power output exhibited a significant main effect for treatment (F = 5.75, *P* = 0.009; ƞ^2^ = 0.307) but not for measures (F = 2.27, *P* = 0.12; ƞ^2^ = 0.14). A significant treatment × measure interaction was found (F = 2.48, *P* = 0.05; ƞ^2^ = 0.16), the condition PP was significantly lower than other conditions at the beginning of the exercise protocol (Fig. 2B).

According to Bayes Factor analyzes for time of TT-test execution, there is a posterior probability of 85.9% and 92.1% to observe a difference between PP and PC and PP and CC, respectively. These Bayes Factor indicated that the posterior probability favoring the alternative hypothesis were moderate and strong, respectively. According to Bayes Factor analyzes for average power output, there is a posterior probability of 96.2% and 99.8% to observe a difference between PP and PC and PP and CC, respectively. These Bayes Factor indicated that the posterior probability favoring the alternative hypothesis were strong and very strong, respectively. The results from inferential and Bayesian statistics are presented in Table 3.

**Table 3:** Bayes factor analysis for time of TT-test execution and average power output among caffeine supplementation conditions.

Experimental condition		Prior odds	Posterior odds	BF_10, U_	Probability %
**Time (s)**					
PP	PC	0.587	3.574	6.084^M^	85.9
PP	CC	0.587	6.889	11.728^S^	92.1
PC	CC	0.587	0.164	0.280^a^	21.9
**Average power output (W)**					
PP	PC	0.587	14.862	25.301^S^	96.2
PP	CC	0.587	47.066	80.125^V^	99.8
PC	CC	0.587	0.103	0.175^a^	14.9

The posterior odds have been corrected for multiple testing by fixing to 0.5 the prior probability that the null hypothesis holds across all comparisons (Westfall, Johnson, & Utts, 1997). Individual comparisons are based on the default *t* test with a Cauchy (0, r = 1/sqrt(2)) prior. The “U” in the Bayes factor denotes that it is uncorrected (PP: Placebo–Placebo; PC: Placebo–Caffeine; CC: Caffeine–Caffeine). Letters indicate the outcome classified as: A = anecdotal; M = moderate; V = very strong; E = extreme favoring the alternative hypothesis; a = anecdotal; m = moderate favoring the null hypothesis.

### HRV at TT-test

**Figure 3:**
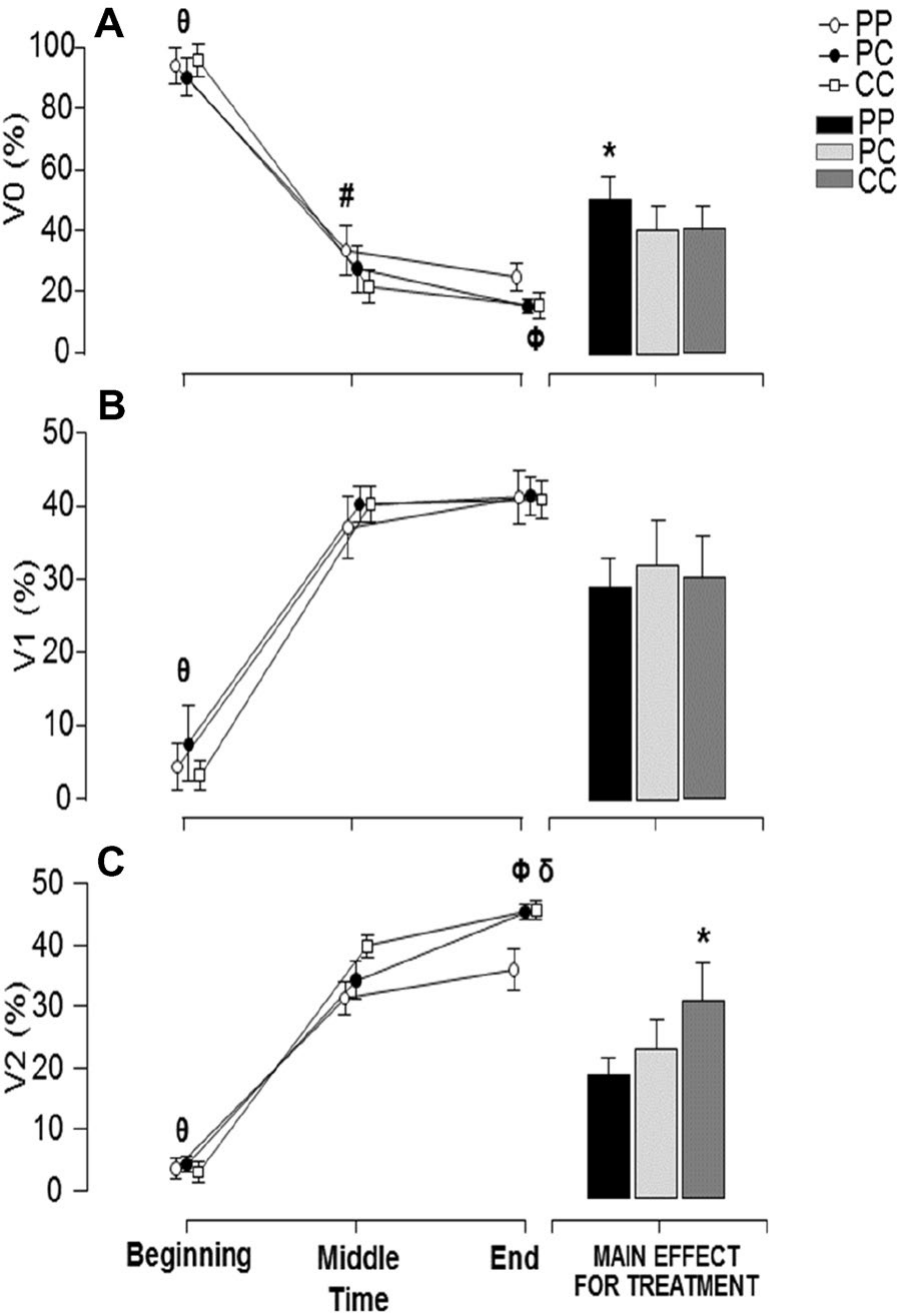
(**A**) Mean ± SE of V0 (%) at the beginning, midway and final of exercise protocol with 3 experimental conditions: Placebo-Placebo (PP), Placebo-Caffeine (PC) and Caffeine-Caffeine (CC). (θ) Beggining was significantly different from midway and final of the exercise protocol in all experimental conditions; (Φ) Midway was significantly different from final of the exercise protocol in PC experimental conditions (*P* < 0.05); (#) Significantly difference between CC and PP experimental conditions at the midway of the exercise protocol (*P* < 0.05); (*) Significantly different from other experimental conditions (*P* < 0.05). (**B**) Mean ± SE of V1 (%) at the beginning, midway and final of exercise protocol with 3 experimental conditions: Placebo–Placebo (PP), Placebo–Caffeine (PC) and Caffeine–Caffeine (CC). (θ) Beggining was significantly different from midway and final of the exercise protocol in all experimental conditions (*P* < 0.05). (**C**) Mean ± SE of V2 (%) at the beginning, midway and final of exercise protocol with 3 experimental conditions: Placebo–Placebo (PP), Placebo–Caffeine (PC) and Caffeine–Caffeine (CC). (θ) Beggining was significantly different from midway and final of the exercise protocol in all experimental conditions (*P* < 0.05); (Φ) Midway was significantly different from final of the exercise protocol in PC experimental conditions (*P* < 0.05); (δ) Significantly difference among PP and both CC and PC experimental conditions at the final of the exercise protocol (*P* < 0.05); (*) Significantly different from other experimental conditions (*P* < 0.05).

The parameter V0 exhibited a significant main effect for treatment (F = 6.77, *P* = 0.005; ƞ^2^ = 0.381) and measures (F = 491.36, *P* < 0.001; ƞ^2^ = 0.978). The parameter V0 was significantly greater in the condition PP when compared to other conditions. The V0 exhibited a decline along the TT-test. The significant treatment × measure interaction was also found for V0 (F = 2.85, *P* = 0.03; ƞ^2^ = 0.206). The condition CC was significantly lower than PP condition at the midway of the exercise protocol. The parameter V1 exhibited a significant difference only for measures (F = 1015.27, *P* < 0.001; ƞ^2^ = 0.989). It was observed a significantly increase along the TT-test (Fig. 3A,B). Regarding the parameter V2, it was found a significant main effect for treatment (F = 8.89, *P* = 0.001; ƞ^2^ = 0.447) and measures (F = 127.30, *P* < 0.001; ƞ^2^ = 0.920). The V2 was significantly greater in the condition CC when compared to other conditions. Additionally, the V2 increased along the TT-test. The significant treatment × measure interaction was also found for V2 (F = 3.54, *P* = 0.01; ƞ^2^ = 0.243). At the condition PC, the V2 exhibited a significantly increase from midway to the final of exercise protocol. At the final of exercise protocol, CC and PC exhibited greater V2 when compared to the condition PP (Fig. 3C).

According to Bayes Factor analyzes, there is a posterior probability of 90.1% and 95.4% to observe a difference in V0 between PP and PC and PP and CC, respectively. These Bayes Factor indicated that the posterior probability favoring the alternative hypothesis were moderate and strong, respectively. For V2, the Bayes Factor analyzes indicated a posterior probability 79.0% and 97.5% to observe a difference between PP and PC and PP and CC, respectively, indicating that the posterior probability favoring the alternative hypothesis were moderate and very strong, respectively. The results from inferential and Bayesian statistics are presented in Table 4.

**Table 4:** Bayesian analysis for the comparison of symbolic analysis parameters among caffeine supplementation conditions.

Experimental condition		Prior odds	Posterior odds	BF_10, U_	Probability %
**V0**					
PP	PC	0.587	5.36	9.13^M^	90.1
PP	CC	0.587	12.20	20.77^S^	95.4
PC	CC	0.587	0.11	0.18^a^	15.2
**V1**					
PP	PC	0.587	0.25	0.42^a^	29.5
PP	CC	0.587	0.12	0.20^m^	16.7
PC	CC	0.587	0.26	0.44^a^	30.4
**V2**					
PP	PC	0.587	2.23	3.76^M^	79.0
PP	CC	0.587	23.00	39.16^VS^	97.5
PC	CC	0.587	0.19	0.32^m^	24.4

The posterior odds have been corrected for multiple testing by fixing to 0.5 the prior probability that the null hypothesis holds across all comparisons (Westfall, Johnson, & Utts, 1997). Individual comparisons are based on the default *t* test with a Cauchy (0, r = 1/sqrt(2)) prior. The “U” in the Bayes factor denotes that it is uncorrected (PP: Placebo–Placebo; PC: Placebo–Caffeine; CC: Caffeine–Caffeine). Letters indicate the outcome classified as: A = anecdotal; M = moderate; V = very strong; E = extreme favoring the alternative hypothesis; a = anecdotal; m = moderate favoring the null hypothesis.

## Discussion

Since our study presented data from (1) procedures to ensure the control of experiments (blood CAF quantification, HRV PRE suppl and POS exerc); (2) performance in TT-test; (3) HRV along and after the TT-test, the discussion was structured to follow the exposed sequence.

In our study, volunteers performed the TT-test under the same nutritional conditions, since the 24-h dietary records did not show significant differences between the PP, PC, and CC conditions. Additionally, the presence of foods, substances, supplements, or medicines containing CAF was also not detected in the records, since blood analysis by HPLC confirmed no serum CAF concentration in athletes at the baseline (for PC and CC conditions), and after the supplementation, when submitted to placebo condition (i.e., PP). As expected, at the conditions PC and CC, blood CAF concentration increased 1 h after supplementation (Table 1). It is important to highlight that the use of analytical methods, as HPLC, to detect CAF in blood samples before and after supplementation is fundamental control method to ensure and validate the supplementation effects^27^ and, in our study, was critical to ensure that volunteers performed the tests exclusively under the effect of experimental supplementation of CAF (i.e., PC or CC conditions) or placebo (i.e., PP condition).

Other adopted procedure to ensure the control of experiments was the recordings of successive RR intervals before and after supplementation, and our results indicated no differences among the CAF (i.e., PC or CC conditions) and placebo condition. A recent study^28^ investigated the influence of different caffeinated beverages (energy drink, coffee, and cola) on HRV parameters obtained through non-linear methods. From a 5-min recording at sitting position, the authors identified that energy drink and coffee beverages could influence the HRV complexity (parameters: Largest Lyapunov exponent and correlation dimension), suggesting a greater variability of successive RR intervals, which have been associated to a better health status. We also used a nonlinear approach to obtain HRV parameters, but chose the symbolic analysis method, since its interpretations is more useful/feasible for clinical practice^28^.

The use of non-linear methods to analyze the RR intervals variability has been reported as a more suitable tool to quantify complex phenomena such as control of cardiac function mediated by ANS^29,30^. With the symbolic analysis method the percentage of occurrence of each pattern of variation was calculated and results are reported as V0%, V1% and V2%. Results from pharmacological blockage^29^ and during tilt test^29^ found that the V0 pattern reflects sympathetic modulation, the V2 pattern parasympathetic modulation, and the V1 pattern reflects sympathovagal balance. Thus, our results support the hypothesis that CAF supplementation (6 mg kg^−1^ of body weight) did not change the sympato-vagal balance at rest, which was valid for recordings at supine position after the TT-test. These results also confirm that the probability of harmful effects from acute CAF supplementation is anecdotal, which was confirmed by Bayesian analysis carried out in our study.

It is important to note that the behavior of successive RR intervals is different at rest and along a high-intensity physical test, then, the significant differences in HRV parameters observed during the TT-test should be discussed apart from data obtained at supine position.

We found a significant acute performance improvement with CAF supplementation, independently of supplementation strategy (i.e., PC or CC) and Bayesian analysis indicated a moderate (PC vs. PP) to strong (CC vs. PP) posterior probability favoring the alternative hypothesis (Table 3). The ergogenic effect of CAF is widely demonstrated, especially in TT-tests^31–33^, as we used.

The present study also observed a better power output performance of athletes in the caffeine condition when compared to the placebo condition. Curiously, the behavior of power output along the TT-test was clearly different between CAF supplementation (PC or CC) and placebo (Fig. 2B). At CAF supplementation conditions the power output was higher since the beginning of TT-test, maintaining quasi-stable along all the exercise, while at placebo condition the power was increasing along the exercise. The excitatory and alerting effects caused by CAF may explain the increased locomotor activity seen at the beginning of the test^34^. Mechanisms involving the effects of caffeine at the level of the central pattern generator of the lumbar spine network, enhancing the locomotor action, have been recently described^35^, facilitating limb activity through inhibition of A1 receptors and subsequent activation of dopamine receptors through an intracellular mechanism dependent on cAMP-dependent protein kinase. This effect probably leads to a great ability to develop power output since the beginning of the exercise.

Compared to tests with anaerobic predominance, as the Wingate test, the chosen TT-test spends a long time to be completed, making it infeasible to apply a maximal power output all time along the exercise. It is expected that experienced cyclists learn to control the intensity to complete the TT-test, then it is interesting the divergent behavior between supplemented conditions and placebo, since at placebo conditions volunteers adjusted the power output along the exercise, while at CAF supplementation they maintained the power output along all TT-test, being higher than placebo at the beginning of TT-test.

In general, well-designed caffeine supplementation strategies can contribute to the improvement of countermovement jump (CMJ) height, average power, peak power, endurance muscle, and strength muscle^36^. Findings^37^ support the hypothesis that the ergogenic effect of CAF would act in a dose-dependent manner. Furthermore, they suggest that habitual consumption of products containing CAF would alter the CAF supplementation effect. Durkalec-Michalski et al.^37^ observed in their study, testing different doses of CAF, that among judokas who usually consumed products containing CAF, only 9 mg kg^−1^ body mass CAF was more effective than 3 mg kg^−1^ body mass. Thus, they suggested that athletes who habitually consume caffeine-containing products would need higher doses to promote specific performance improvement.

The ergogenic effects of caffeine depend on several mechanisms that can vary, such as time course, dose, and magnitude of dependence. Regular caffeine consumption positively induces the production of the Cytochrome P450 (CYP1A2) enzyme group, increasing the rate of metabolism in regular users^5^. Therefore, different consumption strategies added to acute intake doses (pre-test) can produce distinct ergogenic effects. For example, Beaumont et al.^22^ observed that chronic (21 days) use of caffeine supplementation (1.5 mg kg^−1^ day^−1^) followed by acute ingestion (3 mg kg^−1^ body mass of caffeine) induced tolerance to the ergogenic effect. This strategy impacts the ability to perform total work on the cycle ergometer, generating tolerance effects on endurance performance. Furthermore, constant caffeine use also affected cycle ergometer sprint performance. Lara et al.^4^, followed athletes daily using an incremental test and Wingate test in cycle ergometer to test the effects of caffeine supplementation (3 mg kg^−1^ day^−1^) for 20 days. They found that this strategy chronically caused a reduction in the magnitude of ergogenicity in the ventilatory response. However, it significantly increased peak power during a maximal incremental test during the first 15 days of ingestion. They also found an improvement in VO_2_max in the first four days when compared to placebo treatment. Daily caffeine intake before exercise also produced a higher peak power for approximately 18 days after ingestion. After that period, the performance increases were not statistically different from placebo. These results suggest the existence of progressive tolerance to the performance benefits of caffeine, as the ergogenic magnitude of caffeine was verified in the first days of ingestion for both endurance and muscle power exercises. Thus, the time course of habituation is poorly understood and can be mainly affected by routine caffeine consumption. The impact of habituation probably can be modified by acute intake substantially greater than usual.

Concerns about caffeine consumption on cardiovascular activity during intense exercise are commonly related to dose, being reported that high-dose of caffeine intake can cause tachycardia, palpitations, and a rapid rise in blood pressure^38^. However, moderate caffeine intake does not adversely affect cardiovascular health^39^, despite moderate CAF consumption could affect contractility, myocardial conduction, vascular tone, and the sympathoadrenal system^40^. Previous findings^41^ indicated that both 3 and 6 mg kg^−1^ of caffeine were able to increase parasympathetic modulation following an acute bout of anaerobic exercise in recreational athletes. In this setting, the results of this study reinforce the possibility of increasing parasympathetic modulation after caffeine ingestion and add important information suggesting that the relationship between caffeine dosage and parasympathetic reactivity is not linear.

Sarshin et al.^41^ applied a dose window of 3–6 mg kg^−1^ and found an increased resting cardiac autonomic modulation and faster post-exercise autonomic recovery after an anaerobic exercise bout (Wingate test) in recreationally active young men. These data present robust evidence of an interesting clinical effect of CAF, diverging from the mainstream of harmful effect of CAF supplementation on cardiovascular health. It is worthwhile to emphasize that^41^ did not analyzed the RR interval variability along the exercise, leaving a lack of information about the safety of caffeine during intense exercise.

Our data obtained along the TT-test indicated a progressive increase of the parameter V2, achieving higher values at CAF supplementation (PC and CC), when compared to placebo. These find suggest a progressive increase of an indicator of parasympathetic activity^17^.

The pattern variation of successive RR intervals, evaluated by symbolic analysis along the TT-test revealed that the sympathetic modulation (V0) had similar behavior between the conditions (PP, CP, and CC) at the beginning and midway of the exercise, being more pronounced at the beginning of the test, as expected, owing to the high sympathetic drive at the beginning of exercises. The V0 exhibited a decline along with the exercise, which could be related to expected cardiovascular adjusts along the submaximal long exercise. At the final of TT-test, we found an interesting behavior with smaller V0, a marker of sympathetic modulation, at CAF conditions (PC and CC) when compared to placebo. The parameter V1 (pattern reflecting sympathovagal balance) exhibited an increase along the exercise, with similar behavior among the conditions. The parameter V2 (pattern reflecting parasympathetic modulation) increased along the TT-test similarly among supplementation conditions (PP, PC, and CC) at the beginning and midway of the exercise. However, at the final, a prominent parasympathetic modulation was observed in the CAF conditions (PC and CC) compared to the placebo.

These results could reflect a protective effect of CAF during the used TT-test since the sympathetic drive was not the greater in CAF conditions at the beginning of the activity, despite a higher power output at this moment. Autonomic control during physical exercise under CAF conditions can be modified; the baroreflex is attenuated at the brainstem level due to activation of the metaboreflex. Increased metabolic accumulation (i.e., lactate, P_i_, and H^+^) caused by the use of CAF, as a result of increased cellular metabolism, promotes activation of metaboreceptors. As a consequence, stimulation of non-myelinated afferent fibers (III and IV) leads to sympathetic activity, inducing a more accentuated parasympathetic response due to the maintenance of output power for a longer time during exercise^25^. Similar responses were observed by Bunsawat et al. The authors^42^ observed a parasympathetic response with the ingestion of 400 mg of caffeine (capsules) compared to the placebo condition, resulting in greater exercise performance.

Divergently from CAF conditions (PC and CC), the placebo condition demanded more power output to complete the exercise at an expected time (i.e., shorter time as possible) (Fig. 2B), which justifies a more prominent sympathetic drive at the final TT-test. In line with this hypothesis, a parasympathetic withdrawal will be expected at the placebo condition, as we found. This suggests that the rapid onset of the sympathetic response in the final phase (i.e., TT-final) of exercise observed in the placebo (Fig. 3A) condition may have induced an increase in Ca^2+^ release, as well as a decrease in Ca^2+^ reuptake by the sarcoplasmic reticulum, making available more Ca^2+^ to the myocardial contractile mechanism, hindering a more pronounced parasympathetic response^43^.

A direct effect of caffeine could not be neglected since the protective effect of caffeine on the cardiovascular system is reported by Gourine and Gourine^44^, highlighting the importance of neural mechanisms involved in heart protection against lethal ischemia/reperfusion injury. They suggested that effective cardioprotection strategies should increase cardiac parasympathetic activity, thus conferring plausible efficiency in reducing myocardial damage and decreasing myocardial morbidity and mortality^44^. Our data could corroborate with this hypothesis, since CAF (6 mg kg^−1^) induced a greater vagal tone (V2 parameter) and smaller sympathetic activity (V1 parameter) during high-intensity activity, despite it is not possible to attribute this “cardioprotective” effect to a direct action, which is out of scope from our study, being necessary more experiments with animal and/or isolated cells to confirm it. Notwithstanding, it is important to note that the posterior probability of greater V2 and smaller V0 along TT-test with CAF supplementation were moderate to very-strong, as found with Bayesian inference (Table 4).

Clark et al.^45^ did not find differences in RMSSD, a HRV parameter obtained with a linear method (time domain approach) and associated with parasympathetic modulation. However, three methodological aspects need to be emphasized when comparing the results from this study^45^ and ours. They used (1) a low dose of CAF (an energy drink formula containing 140 mg of caffeine); (2) a graded exercise test to exhaustion; (3) a linear method to obtain estimations of heart autonomic modulation. A significant parasympathetic withdrawal is expected in exercise designed to increase the intensity in a predetermined manner until failure, and the lower CAF dose could be not sufficient to sustain the parasympathetic modulation along the exercise. Our study used an exercise mimetizing a competition, where the athlete could choose the intensity, but be aware of the aim of the exercise (i.e., conclude the 16 km as fast as possible). The last relevant difference between^45^ and our study was the chosen method to analyze the variability of successive RR intervals, and nonlinear methods, as we used, is reported to be more suitable to quantify complex phenomena such as control of cardiac function mediated by ANS^46–48^.

Despite the great effort to control the variables involved in the study, some limitations should be considered. One of the limitations of our findings is associated with the use of a cycle ergometer in the laboratory. Thus, the findings of the present study should be confirmed in additional research protocols, which use field tests (i.e., cycling competition or simulations) or assessments using the athlete's bicycle, to accurately transfer the results of this investigation to coaches. Other limitations of the present study are related to the analysis of the plasma catecholamine concentrations and the sympathetic nerve activity; however, we used HRV, a simple non-invasive method and one of the most promising quantitative markers of autonomic heart rate balance^49^. The urine content of caffeine metabolites (paraxanthine, theobromine, and theophylline) was also not analyzed in the present study, which could indirectly reflect the pharmacokinetics of CAF. It has been shown that the increase in the concentration of paraxanthine (one of the metabolites of CAF) has a different relationship with plasma levels of CAF after the 60-min period of CAF ingestion (time used in the present study)^50^. The increase in paraxanthine occurs at a slower rate than the CAF in plasma during this period (60 min), making its detection in urine even more difficult^51^. Finally, we do not control Cytochrome P450 (CYP1A2) polymorphisms. These variations in genes encoding CYP1A2 proteins can impact caffeine metabolism and potentiate dopaminergic neurotransmission. However, as this condition is uncommon, conducting a study with this genetic outcome requires a robust number of participants^52^.

As a novelty from our study we highlight the use of a 16 km TT-test, a long task that mimetize cycling competitions tasks, instead of predominantly anaerobic tests, which have been used in previous studies, investigating the cardiovascular effects of CAF supplementation. The use of symbolic analysis to estimate sympathovagal modulation, and the Bayesian inference as a statistical approach, also represent novelty, since previous studies in the field of our study did not include these promising mathematical approaches.

In conclusion, our study has provided evidence that acute intake of CAF (6 mg kg^−1^) promotes ergogenic effects in cyclists during 16 km TT-test, enhancing their average power output at the beginning of the TT-test and sustaining it along the exercise. In addition, supplementation or withdrawal of CAF (6 mg kg^−1^) for 4 days proved to be safe ergogenic strategies, since heart autonomic balance did not change 60 min after acute CAF supplementation, 10 min after the TT-test and, demonstrated a relevant cardioprotective effect, through increased vagal tone, along the TT-test, with a good posterior probability estimated by Bayesian inference. It is noteworthy that the cardioprotective effect of caffeine was observed in healthy volunteers, and our findings should not be transmitted to patients at high cardiovascular risk or cardiovascular disease. Other studies must be carried out to assess the effects of different doses of CAF, and establish the most accurate dosage that enhances the results, favors cardioprotection, and minimizes risks.

## Methods

### Sample size

A statistical analysis of the test's power (G * Power 3.1) was performed to estimate the sample size based on the time trial cycling test (16 km) of a pilot study (n = 8 cyclists). Assuming repeated measures, within factors (three interventions). The projected sample size was n = 12 with an effect size “f” of 0.41, alpha = 0.05 and power = 0.80. Using a more conservative approach (a priori), with an effect size “f”of 0.36 (average effect size), the projected sample size was n = 14, alpha = 0.05 and power = 0.80.

### Subjects

Therefore, fourteen (n = 14) male recreationally-trained cyclists participated in an information session with anamnesis purpose before the study began. The inclusion criteria were: all had at least 4 years of experience, participated in at least 20 competitions (in 2018 and 2019), have no history of cardiorespiratory, gastrointestinal, and musculoskeletal disorders in the last 3 months. A simple questionnaire evaluated the training volume of all participants to warrant a homogenous sample for the study. They had a mean ± standard deviation (SD) age of 34.1 ± 4.4 years, a height of 178 ± 9 cm, body mass of 79.1 ± 11.8 kg, body mass index of 24.6 ± 2.1 kg m^2^, maximal oxygen uptake (VO_2_max) of 51.5 ± 6.3 mL kg^−1^ min^−1^, and power output max (Wmax) of 398.9 ± 35.1 W. The training volume of the cyclists was 202 ± 83 km per week. In addition, a validated caffeine consumption questionnaire was administered to the participants, showing that all participants were moderate to high caffeine consumers (285.92 ± 108.04 mg day^−1^)^53,54^. The research protocol (2.540.958/2018) was approved by the Research Ethics Committee of the Federal University of Rio de Janeiro (UFRJ), and that all methods were performed according to the guidelines of the Declarations of Helsinki. The project was registered with the Brazilian Registry of Clinical Trials (RBR-5745nv—04/12/2019), which is accredited by the World Health Organization.

## Study design

A randomized, double-blind, crossover, placebo-controlled design was used in this study. On the first visit to the laboratory, participants underwent dietary assessment and cycling test to exhaustion. On the second visit, cyclists became familiar with the time trial test. So they received four visits from the researchers at home, one per day, for delivery and verification of capsule consumption (according to randomization). The other day they consumed the capsule acutely 60 min before the 16 km time trial test. The study preconized a seven-day washout between the different intervention strategies^19–21,55^. We tested the following strategies: Placebo–Placebo (PP), participants received Placebo (4-day supplementation), and Placebo (acute ingestion, 60 min before simulated cycling TT-test completed). Placebo capsules were 250 mg of magnesium silicate single daily dose. Placebo–Caffeine (PC), participants received Placebo (4-day supplementation), and Caffeine (acute ingestion, 60 min before simulated cycling TT-test completed). Caffeine capsules were 6 mg kg^−1^ body mass. Caffeine–Caffeine (CC), participants received Caffeine (4-day supplementation), and Caffeine (acute ingestion, 60 min before simulated cycling TT-test completed). At the first visit to the laboratory, the researchers verified the routine energy and caffeine intake of food, VO_2_max, and workload capacity in the graded test until exhaustion in the cycle ergometer. The athletes were instructed to withdraw all their caffeine consumption (i.e., food sources of caffeine) during the experiment and were monitored by telephone contact, email, and in-person. On the test day, cyclists arrived fasting in the laboratory, and soon an intravenous cannula (20G Jelco; B. Braun Medical Inc., Bethlehem, PA, USA) was inserted into the forearm, and then two blood samples (10 mL) were obtained: before ingestion of capsules (baseline) and 60 min after intake of capsules. Cyclists did not exercise 24 h before the experimental trials in the laboratory. The athletes were instructed to continue the routine of daily training. The experimental trials were performed at the same time of day (7:00 a.m.).

### Informed consent

Informed consent was obtained from all the participants involved in the study.

## Methodology

### Randomization

We use the block randomization method to ensure a balanced sample size across the groups using an online page http://www.randomization.com (Supplementary File-Disposition of study participants).

### VO_2_max and workload capacity

To determine the VO_2_max, participants performed a graded exercise test to exhaustion on the cycle ergometer (Cefise, São Paulo, Brazil). Participants started pedaling with 113 W power, with 45 W increments every 2 min, and the pedaling rate 88 rev min^−1^ was kept constant until exhaustion. The maximum workload capacity (Wmax) was determined by the following equation: Wmax = Output power (output) + ((t/113) * 45), “Output” is the workload in the last completed stage and “t” Is the time spent in the final stage not completed^53^. Heart rate was monitored continuously (Polar Electro Oy, Kempele, Finland). The pulmonary gas exchange was determined breath by breath for carbon dioxide, oxygen concentrations, and minute ventilation using a VO2000 gas analysis system (MedGraphics, St. Paul, MN, USA). The equipment was automatically calibrated according to the manufacturer's specifications before each test. The present study determined and validated the VO_2_max following these criteria: increase in VO_2_ less than 2.1 mL kg^−1^ min^−1^ by increasing the intensity; exhaustion of the individual; the respiratory exchange ratio bigger than 1.10. The plateau in VO_2_ was determined when the difference in oxygen consumption in the final 30 s of the last two stages (∆VO_2_) was ≤ 2.1 mL kg^−1^ min^−1^.

### Nutritional intervention

The participants took a dose of anhydrous caffeine (6 mg kg^−1^ body mass) or placebo (250 mg magnesium silicate), provided in gelatin capsules, identical in color, size, and appearance. Because the participants are used to consuming average amounts of caffeine daily, around 285.92 ± 108.04 mg day^−1^ (verified through a questionnaire), the present study chose to offer a higher dose than usual. This dose (6 mg kg^−1^ of body weight) represented a dosage of 474.78 ± 70.80 mg, exceeding the average amount of usual consumption, which made it possible to verify the effects of tolerance. In the presence of a researcher, all athletes were instructed to take a single capsule daily at the same time (9:00 a.m.) during the 4-day supplementation. In acute ingestion, the capsule was administered with 250 mL of water before simulated cycling TT-test completed. Supplements for each participant were prepared and separated by a non-affiliated researcher to ensure double-blinding.

### Blinding efficacy

Both in the caffeine and the placebo trials, after the exercise session, participants responded to the following question: “Which supplement do you think you have ingested?”. The question had three possible responses: (a) “caffeine”, (b) “placebo” and (c) “I do not know”^56^. With this information, we would know the percentage of belief of caffeine consumption. A high frequency of this belief could influence the study results as described by other authors^56^.

### Habitual food intake recording and caffeine-containing foods

Cyclists were instructed to maintain their dietary and hydration patterns. A 24-h dietary record was completed by each athlete on the first visit and, before the first test, it was photocopied and returned to the athletes so that the same diet could be repeated for subsequent trials. The energy intake, carbohydrates, total proteins, and total lipids was determined. The TACO database was used to quantify macronutrient intake and the Dietpro 5i software (version 5.8.1, Dietpro, Minas Gerais, Brazil; https://dietpro.com.br/store/) for calculating nutrients and ensuring control of the study. To assess the dietary frequency and the amount of caffeine, a validated questionnaire was applied by trained nutritionists. The questionnaire consists of a list of dietary sources of CAF (coffee, tea, cocoa, chocolate, soft drinks, medicines, and dietary supplements) and the time of consumption. Household measures were used to assess the amount of food consumed according to the following frequency of consumption^54^. Types of foods, dietary supplements, and medications that contained caffeine were identified. The CAF content was obtained from the U.S. DEPARTMENT OF AGRICULTURE food composition databases, on food labels, and the medication package insert.

### HRV analysis

Participants went to the laboratory with a comfortable temperature (20–23 °C) at standardized times, individually scheduled on an 8-h fast and after a good night’s sleep (at least 6 h). Successive RR intervals were acquired for 5 min while supine (REST) and along the TT-test. Beat-to-beat intervals were recorded by a validated heart rate monitor (Polar RS800, Polar Electro Oy, Kempele, Finland) for HRV analysis^57^. The sampling frequency was set at 1000 Hz, the smoothness prior method with alpha set at 500 was used for detrended the R-R intervals series. They were studied 48 h far away from the last bout of physical exercise to avoid the short-term autonomic and cardiovascular confounding after-effects induced by recent training sessions. Recordings at supine position (HRV-SP) were performed three times, before the ingestion of the capsule (PRE suppl), 60 min after supplementation (PRE exerc), and 10 min after the TT-test (POST). Recording of the HRV during the TT-test (HRV-TT) was done during the entire time of the exercise execution. The nonlinear dynamics of successive RR intervals were assessed by symbolic analysis^29^ during the TT-test. The R-R dynamics were classified into three pattern families: (I) patterns with no variation (V0; all three symbols were equal); (II) patterns with one variation (V1; two consequent symbols were equal, and the remaining symbol was different); and (III) patterns with two unlike or like variations (V2; all of the symbols were different from the previous ones). The percentage of the patterns V0 was calculated as sympathetic modulation predominance, V1 reflects sympathetic and parasympathetic modulation, and the V2 calculated as a parasympathetic modulation as suggested by Santos et al.^17^. The data (i.e., successive RR intervals) were transferred to a computer for analysis. For the recordings from supine position, the 5-min successive RR intervals were selected for analysis, while for TT-test, the visual inspection was carried out to find and select a segment of 1000 successive RR intervals where the stationarity of the time series was acceptable at three moments: beginning, midway and final of each TT-test session. All RR interval variability analyses were carried out by the same researcher which was blind for the applied treatment in each data set.

### Blood caffeine levels

The measurement of blood levels of caffeine was performed at baseline and 60 min after intake of capsules. Serum was obtained by centrifugation at 2.500 rpm at 4 °C for 20 min. The resultant serum was stored at − 20 °C until the analyses could be performed. The caffeine blood levels were determined using a HPLC method previously described^20^. The HPLC analyses were carried out using a Shimadzu chromatograph (Shimadzu Corp., Kyoto, Japan).

### 16 km time trial test (TT-test)

**Figure 4:**
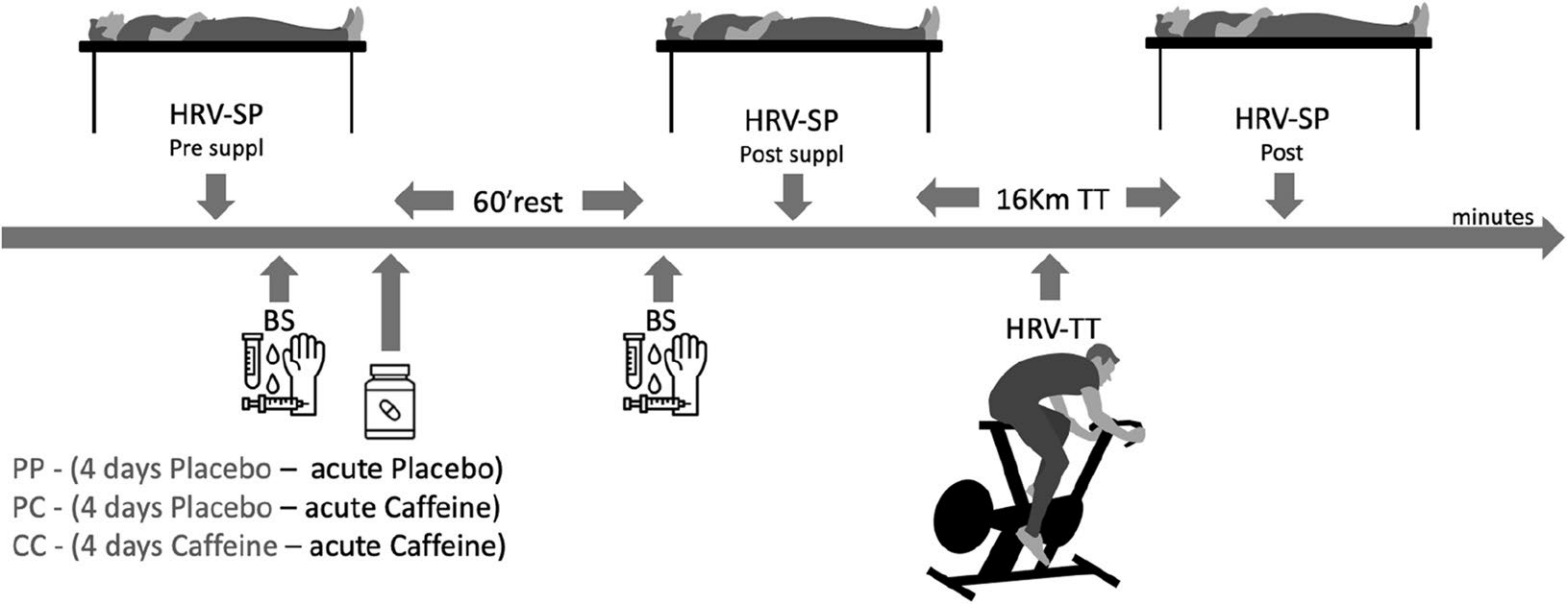
Dynamics of data acquisition. Placebo–Placebo (PP), Placebo–Caffeine (PC) and Caffeine–Caffeine (CC) conditions were tested in 16 km time Trial (16 km TT) performance and HRV analysis were done pre supplementation (HRV-SP PRE suppl), post supplementation (HRV-SP POST suppl) during 16 km TT (HRV-TT) and post 16 km TT (HRV-SP Post).

Before the TT-test, the participants underwent a 5-min warm-up at 113 W (88 rev min^−1^), followed immediately by the TT-test. The protocol consisted of a continuous test, the participant was instructed to cover the distance of 16 km in the work intensity as quickly as possible with 50% of the maximum workload capacity (199.67 ± 17.90 W equivalent ~ 407 kJ) on the cycle ergometer^58^. The cycle ergometer was connected to a laptop using the “Ergometric” software (version 7.0, Cefise, São Paulo, Brazil; https://cefise.com.br/produtos/cicloergometria/) for the collection and storage of data, such as power (W) and cadence (rev min^−1^). Participants did not receive any performance feedback during the tests. The only information that the participants received was the distance reached: 2 km, 4 km, 6 km, 8 km, 10 km, 12 km, 14 km, and 16 km. The analyses of the power outputs were measured in 3 equals intervals (Beginning, Midway and Final) of the completed TT-test time curve. All tests were carried out in a laboratory with a controlled temperature of 20–23 °C with a relative humidity of 45–55%. The dynamics of data acquisition in the laboratory can be seen in Fig. 4.

### Statistical analysis

Data expressed as mean ± SD (mean ± SE in graphics) or median (interquartile deviation) according to the normality assessed by the Shapiro Wilk test. When data showed normal distribution the variables were analyzed by mixed ANOVA with repeated measures. The Mauchly sphericity test was performed for all tested variables, and the Greenhouse–Geisser correction was used in cases where the sphericity assumption was violated. Tukey's post hoc test was used for means multiple comparisons. When data presented a non-normal distribution the Kruskal Wallis test was used (version 19.0, SPSS; https://www.ibm.com/br-pt/analytics/spss-statistics-software). Effect sizes (ES) were reported as η^2^ p with 95% confidence intervals and Delta (Δ) of CAF supplementation was calculated as the difference between the means corresponding to CAF (PC and CC) and placebo (PP). For all statistical analyzes, a significance level of 5% was adopted (α = 5%). To check the qualitative outcomes and the probability to replicate the same results (i.e., the magnitude of the evidence), we applied the Bayes Factor hypothesis testing analyses. The posterior odds have been corrected for multiple testing by fixing to 0.5 the prior probability that the null hypothesis holds across all comparisons^59^. Individual comparisons are based on the default *t* test with a Cauchy (0, r = 1/sqrt(2)) prior. The "U" in the Bayes factor denotes that it is uncorrected. The outcomes were classified as anecdotal (BF10 = 1–3), moderate (3–10), strong (10–30), very strong (30–100) and extreme (> 100) favoring the alternative hypothesis; or anecdotal (BF10 = 1–0.33), moderate (0.33–0.1), strong (0.1–0.03), very strong (0.03–0.01) and extreme (< 0.01) favoring the null hypothesis (Lee and Wagenmakers’ classification)^60^. To calculate the probability to find the same results again, we divided the actual BF10 value by BF10 + 1. We made all BF analysis through the JAMOVI.

## Supplementary Information

Supplementary Information 1.

## Abbreviations

CAF: Caffeine
A1, A2a, A2b: Adenosine receptors
cAMP: Cyclic adenosine monophosphate
cGMP: Cyclic guanosine monophosphate
RR: Intervals time elapsed between two successive R-waves
HRV: Heart rate variability
ANS: Autonomic nervous system
V0: Sympathetic modulation
V1: Sympathetic and parasympathetic modulation
V2: Parasympathetic modulation
TT-test: 16 Km time trial test
PRE suppl: Pre supplementation (before the ingestion of the capsule)
PRE exerc: Pre exercise (60 min after supplementation)
POST: Post exercise (10 min after the TT-test)
PP: Placebo–placebo
PC: Placebo–caffeine
CC: Caffeine–caffeine
REST: 5 Minutes while supine
HRV-SP: Recordings of the HRV in supine position
HRV-TT: Recording of the HRV during the TT-test
HRV-SP PRE suppl: Recordings of the HRV in supine position pre supplementation
HRV-SP POST suppl: Recordings of the HRV in supine position post supplementation
HRV-SP Post: Recordings of the HRV in supine position post 16 km TT
CYP1A2: Cytochrome P450
VO_2_max: Maximal oxygen uptake
VO_2_: Oxygen uptake
