# Supplementary figures and images for: Caffeine increases performance and leads to a cardioprotective effect during intense exercise in cyclists
Source: Sci Rep. 2021 Dec 21;11:24327. doi: 10.1038/s41598-021-03158-2 (PMC8692308; doi:10.1038/s41598-021-03158-2)

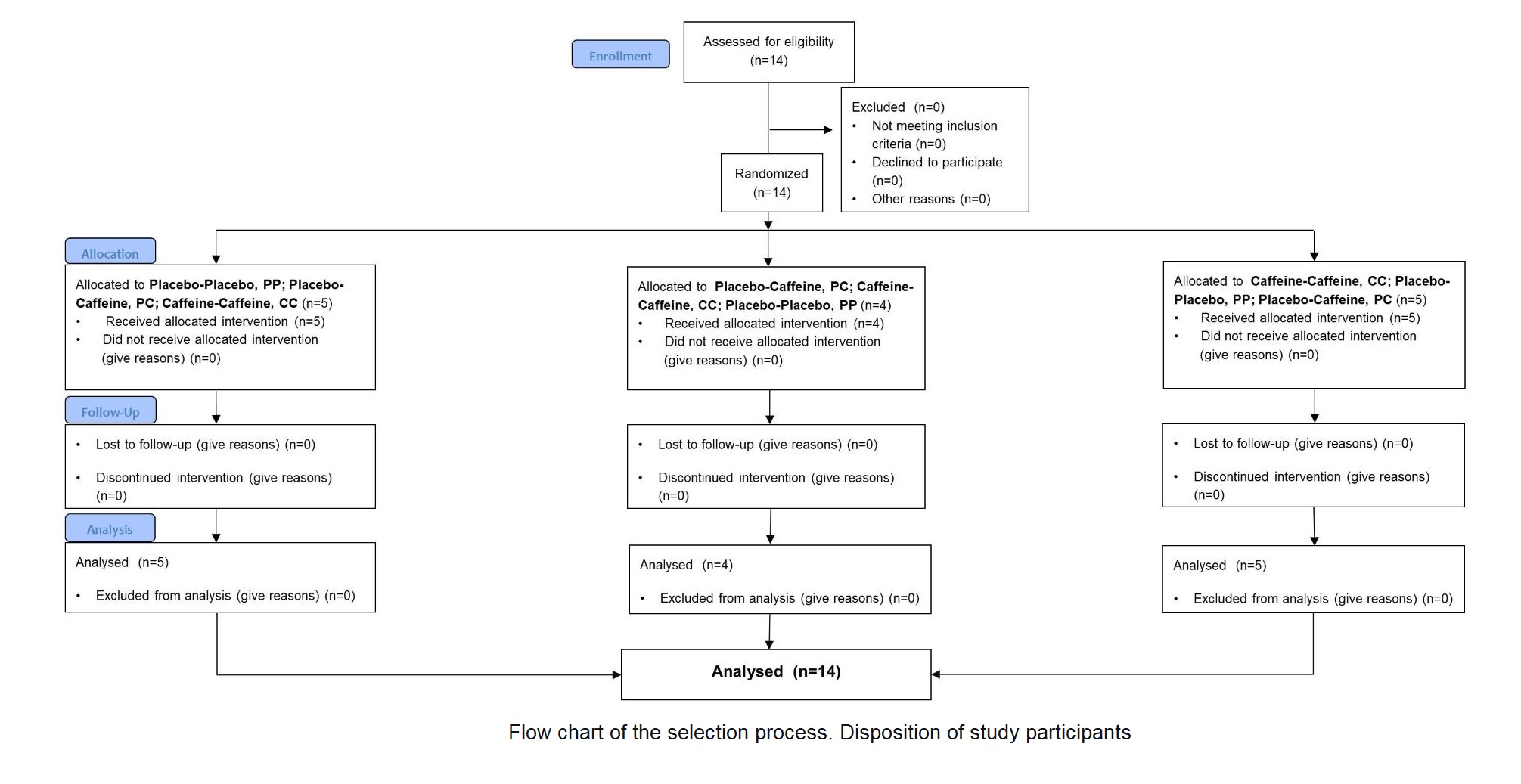

Supplement: Supplementary file 1 — Supplementary Information 1. [file 41598_2021_3158_MOESM1_ESM.jpg]
